# A Rare Presentation of Duodenal Diverticulum Causing Obstructive Jaundice: Lemmel's Syndrome

**DOI:** 10.7759/cureus.33702

**Published:** 2023-01-12

**Authors:** Priyal Shrivastava, Vadlamudi Nagendra, Amruta Varma, Sushma S, Anna Mary Jose

**Affiliations:** 1 Radiology, Jawaharlal Nehru Medical College, Datta Meghe Institute of Higher Education and Research, Wardha, IND; 2 Surgery, Madras Medical College, Chennai, IND; 3 Surgery, Jawaharlal Nehru Medical College, Datta Meghe Institute of Medical Sciences, Wardha, IND

**Keywords:** periampullary diverticulum, duodenal diverticulum, ct, obstructive jaundice, lemmel’s syndrome

## Abstract

Lemmel syndrome is an uncommon pancreaticobiliary consequence of duodenal diverticula. We herein present a case of an 80-year-old male who presented with upper abdominal discomfort. Based on lab values and relevant clinical history, a diagnosis of obstructive jaundice was made. A contrast-enhanced CT scan of the abdomen revealed gross dilatation of intra-hepatic and extra-hepatic bile duct, cystic duct, common bile duct, major and minor pancreatic duct. A contrast-filled outpouching was seen from the medial wall of the second part of the duodenum with duodenal diverticulum and papilla within it. The abrupt termination of the common bile duct and main pancreatic duct adjacent to the thickened wall of the diverticulum was the cause of the patient’s pancreaticobiliary obstruction. In the absence of cholelithiasis or tumor, the duodenal diverticulum that manifests as obstructive jaundice is known as Lemmel syndrome. Prompt identification of Lemmel syndrome can avoid dangerous complications and unnecessary investigations. Gallstones, cholangitis, and bile duct stones are more common in patients with duodenal diverticula. Treatment depends on patient presentation and may involve conservative management, surgical procedures in the form of excision of the diverticulum, or even endoscopic sphincterotomy or stenting.

## Introduction

The most considered causes of obstructive jaundice include choledocholithiasis and biliary/periampullary tumors. However, it is important to be aware of uncommon differential diagnoses to ensure adequate management [1]. Lemmel's syndrome should be investigated as a differential diagnosis for obstructive jaundice when choledocholithiasis or pancreaticobiliary/periampullary tumors are absent. [2]. Named after Dr. Gerhard Lemmel in 1934, the possible existence of a periampullary diverticulum (PAD) could be the likely culprit of Lemmel's syndrome [2,3]. Periampullary diverticula (PAD) can be explained as extraluminal outpouchings of the duodenum that originate within 2-3cm from the ampulla of Vater [4]. Such outpouchings are also known as peripapillary or paravaterian diverticula, in addition to being called PAD [5]. The incidence of PAD ranges from 1% to 27% [6]. Although mostly asymptomatic, underreporting of symptomatic duodenal diverticula using imaging modalities is a prevalent issue mostly caused by the lack of pathognomonic signs and symptoms [7]. The primary diagnostic tools are ERCP or EUS, and non-invasive testing like CT scans support the diagnosis [3].

## Case presentation

An 80-year-old man diagnosed with chronic kidney disease came to the hospital complaining of gradually progressive yellowish discoloration of sclera, dark yellow urine, constant non-radiating dull aching pain in the epigastric region of the abdomen, and nausea for seven days. He had no complaints of itching, fever, vomiting, hematemesis, melena, haematochezia, or weight loss. His past surgical history was insignificant for any major operations or hospital stay. He also complained of similar episodes over the past year that would resolve in 3-4 days without any treatment. He was non-alcoholic, non-hypertensive, and non-diabetic. 

Upon physical examination, his vitals were stable, and he had yellowish discoloration of the sclera. Tenderness could be elicited in the epigastric region and the right upper quadrant. Murphy’s sign was found to be equivocal. His laboratory workup showed elevated total serum bilirubin levels (29 mg/dl), with direct bilirubin accounting for most of it (18mg/dl). ALP (815 U/L), AST (180 U/L) and CRP were also found to be raised above normal values. The rest of the lab investigations fell within normal limits. A contrast enhanced-CT scan of the abdomen was performed for further evaluation.

Imaging findings

**Figure 1:**
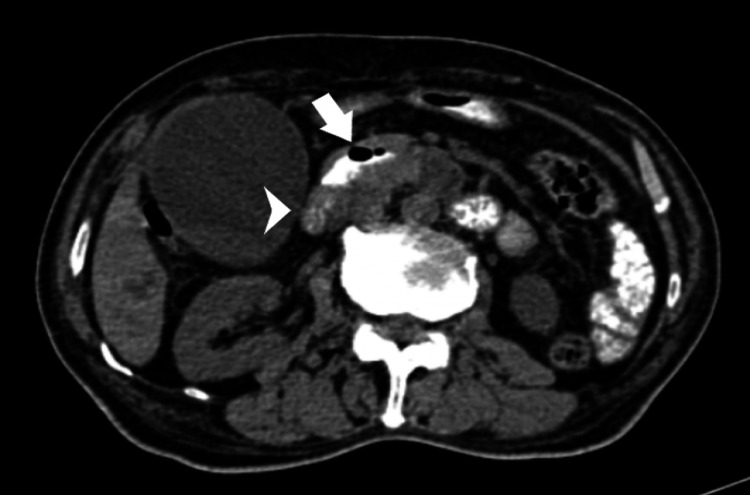
Axial oral and intravenous contrast-enhanced Computed Tomographic section at the level of gall bladder showing pooling of oral contrast in the diverticulum with air (arrow) arising from the medial wall of the second part of duodenum. Arrowhead showing the normal distal second part of the duodenum.

**Figure 2:**
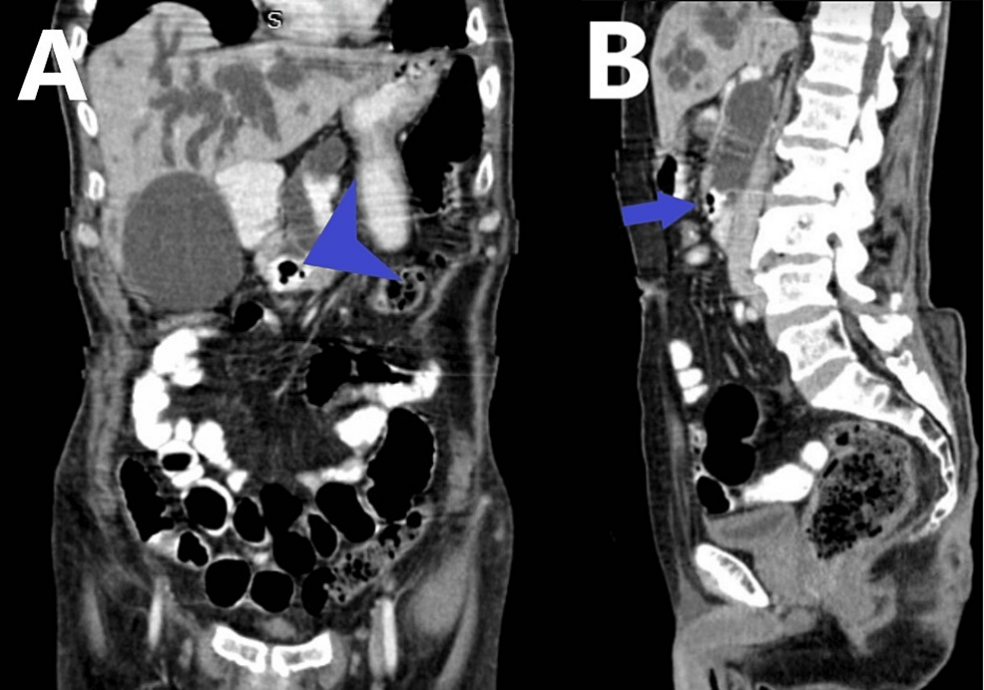
Coronal (A) and sagittal (B) oral and intravenous contrast enhanced multiplanar reformatted computed tomographic images of abdomen showing diverticulum (arrowhead and arrow)

Contrast-enhanced CT examination of the abdomen revealed a contrast pooled duodenal diverticulum (Figures 1, 2) measuring 2.7 x 1.5 cm from the medial wall of the D2 segment of the duodenum.

**Figure 3:**
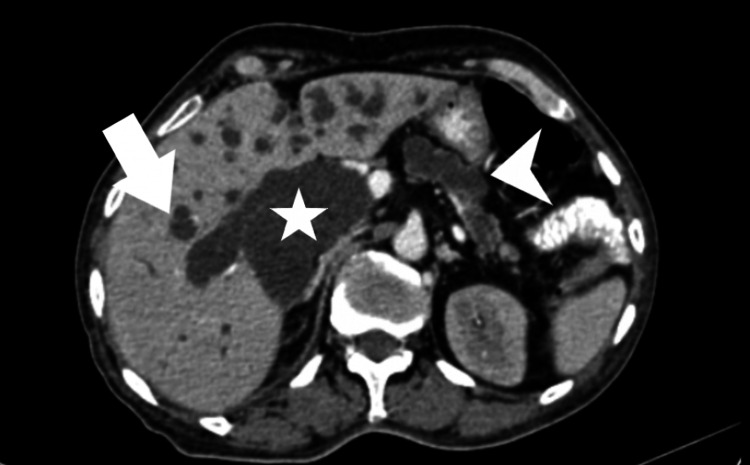
Oral and intravenous contrast-enhanced computed tomographic axial section of the abdomen at the level of the liver showed grossly dilated intrahepatic biliary radicles (arrow), grossly dilated common bile duct (asterisk), and dilated main pancreatic duct (arrowhead).

The diverticulum is causing gross dilatation of the intra-hepatic biliary radicals, common hepatic duct, common bile duct, and main pancreatic duct with abrupt termination in the distal course (Figure 3). Asymmetric heterogeneously enhancing wall thickening of the diverticulum is seen with the abrupt termination of the common bile duct and main pancreatic duct adjacent to the thickened wall. The entire pancreas appeared atrophic with the grossly dilated entire main and minor pancreatic duct.

Treatment and outcome

The patient was advised by medical management first to bring the bilirubin levels to normal. Endoscopic retrograde cholangiopancreatography (ERCP) was tried, but the stent failed to be inserted. The patient later underwent surgical excision of the diverticulum. He was discharged one week later with a good recovery. On follow-up six months later, he remained well and had no complaints. 

## Discussion

Lemmel's syndrome is explained as biliary obstruction brought on by an affected periampullary duodenal diverticulum [8]. Lemmel’s syndrome was diagnosed in light of recurring clinical features of biliary obstruction and definitive imaging findings of the abrupt termination of the common bile duct and main pancreatic duct adjacent to the duodenal diverticula with a thickened wall. Diverticula are sac-like protrusions of the entire or partial gut wall that can appear anywhere along the gastrointestinal tract [9]. It is challenging to determine the true prevalence of duodenal diverticula; they are observed in 1%-6% of upper gastrointestinal contrast investigations, 12%-27% of endoscopic studies, and 15%-22% of autopsies. Increasing age, weakening intestinal smooth muscles over time, and rising intraduodenal pressure may all promote the duodenum's outpouching [10]. Diverticula are most frequently found in the duodenum, second only to the colon, in the gastrointestinal tract. The pathophysiology of DD is elusive, although, in the lack of an appropriate muscular sheath or heterotrophic pancreatic tissue, it might manifest as a locus minoris resistentiae, which arises where blood vessels, biliary and pancreatic ducts penetrate through into the wall of the duodenum. The two types of DD are primary or true diverticula and secondary or false diverticula. The secondary ones, which are the result of protracted duodenal ulceration, are referred to as pre-stenotic diverticula. Furthermore, diverticula in the duodenum can also be categorized as intraluminal or extraluminal. The most prevalent type is the false extraluminal type [11].

Duodenal diverticula (DD) are referred to as periampullary, peripapillary, or paravaterian diverticula (PAD) when they develop within 2-3 cm of the ampulla of Vater [5]. It can be divided into three categories depending on where the papilla is in relation to the diverticulum. The major papilla is situated inside the diverticulum in Type I, which is the most prevalent. The major papilla in Type II is found in the diverticulum's margin, while Type III is located close to the diverticulum [12]. Ours was type 1 duodenal diverticula as the major papilla was situated within the diverticula. Lemmel syndrome can occur along one of three different paths of etiology. First, direct mechanical stimulation of PAD may result in persistent ampulla inflammation, which then induces papilla fibrosis. Second, PAD could render the sphincter of Oddi dysfunctional. Third, PAD can mechanically compress the distal common bile duct or ampulla, as it did in our patient [13]. As reported by other studies [7,9], the age range of affected patients was close to 80 years, which is similar to our case. Lemmel's syndrome was observed to have no gender preference [2,7,9,12]. Though not existing in our patient, three separate investigations found a history of surgical intervention in the form of cholecystectomy, which raises the possibility that prior cholecystectomy may be a risk factor for the onset of Lemmel's syndrome [2,9,12].

The diagnosis of this syndrome necessitates imaging studies, including CT, magnetic resonance cholangiopancreatography (MRCP), and barium studies [14]. However, the gold standard diagnostic technique is a side-viewing endoscope during ERCP [13]. On unenhanced CT scan and MRCP, PAD may appear as thin-walled outpouching on the wall of the second and third portion of the duodenum containing gas collection. This collection of gas material may occasionally be filled with fluid, leading to a false diagnosis of a metastatic lymph node. Oral and/or intravenous contrast material helps diagnose PAD accurately [7]. When it is large and fluid-filled, it may also be mistaken for a pancreatic pseudocyst [10].

The majority of these periampullary diverticula are asymptomatic and only occasionally cause complications that are either pancreaticobiliary or non-pancreaticobiliary. Diverticulitis, perforation, hemorrhage, and fistula formation are examples of non-pancreaticobiliary complications. Obstructive jaundice, acute pancreatitis, recurrent gallbladder or bile duct stones, and ascending cholangitis are a few examples of pancreaticobiliary complications [9]. The pancreatic atrophy observed in our patient could be explained by an isolated pancreatic obstruction producing recurring episodes of acute pancreatitis and pancreatic atrophy. This obstruction could be caused by choledocholithiasis and sludges caused by stasis, stenosing papillitis brought on by intermittent episodes of diverticulitis, or intra-diverticular enterolith [15]. In asymptomatic patients, no treatment is suggested. In cases of perforation, conservative management involves nasogastric decompression and broad-spectrum antibiotic therapy [7].

Excision of the diverticulum is a surgery that is required when there is biliary obstruction; it is complex and also linked to higher mortality and morbidity rates. In high-risk cases, endoscopic sphincterotomy or stenting are suitable alternatives [14].

## Conclusions

When choledocholithiasis and/or pancreatic head lesions are absent, Lemmel's syndrome should be evaluated as an uncommon cause of obstructive jaundice. Contrast-enhanced CT is the optimum imaging method for a quick, non-invasive, and accurate assessment of PAD related to Lemmel syndrome. The distal common bile duct or ampulla may be mechanically compressed by periampullary diverticula, resulting in Lemmel's syndrome. The management strategies may change depending on the pathophysiology, mechanism, and underlying symptoms.
